# A concept for holistic whole body MRI data analysis, Imiomics

**DOI:** 10.1371/journal.pone.0169966

**Published:** 2017-02-27

**Authors:** Robin Strand, Filip Malmberg, Lars Johansson, Lars Lind, Magnus Sundbom, Håkan Ahlström, Joel Kullberg

**Affiliations:** 1 Division of Radiology, Department of Surgical Sciences, Uppsala University, Uppsala, Sweden; 2 Centre for Image Analysis, Department of Information Technology, Uppsala University, Uppsala, Sweden; 3 Antaros Medical AB, BioVenture Hub, Mölndal, Sweden; 4 Department of Medical Sciences, Uppsala University, Uppsala, Sweden; 5 Department of Surgical Sciences, Uppsala University, Uppsala, Sweden; Linköping University, SWEDEN

## Abstract

**Purpose:**

To present and evaluate a whole-body image analysis concept, Imiomics (imaging–omics) and an image registration method that enables Imiomics analyses by deforming all image data to a common coordinate system, so that the information in each voxel can be compared between persons or within a person over time and integrated with non-imaging data.

**Methods:**

The presented image registration method utilizes relative elasticity constraints of different tissue obtained from whole-body water-fat MRI.

The registration method is evaluated by inverse consistency and Dice coefficients and the Imiomics concept is evaluated by example analyses of importance for metabolic research using non-imaging parameters where we know what to expect.

The example analyses include whole body imaging atlas creation, anomaly detection, and cross-sectional and longitudinal analysis.

**Results:**

The image registration method evaluation on 128 subjects shows low inverse consistency errors and high Dice coefficients. Also, the statistical atlas with fat content intensity values shows low standard deviation values, indicating successful deformations to the common coordinate system.

The example analyses show expected associations and correlations which agree with explicit measurements, and thereby illustrate the usefulness of the proposed Imiomics concept.

**Conclusions:**

The registration method is well-suited for Imiomics analyses, which enable analyses of relationships to non-imaging data, e.g. clinical data, in new types of holistic targeted and untargeted big-data analysis.

## Introduction

Clinical imaging modalities such as Magnetic Resonance Imaging (MRI), Positron Emission Tomography (PET) and Computed Tomography (CT) have developed rapidly, and it is now possible to image the entire human body in seconds or minutes. Despite the very strong development on the hardware side, the quantitative image analysis methods used today do not utilize the full potential of the collected image data. Whole-body image datasets contain huge amounts of spatially detailed morphological, functional, and metabolic information; a whole-body scan typically contains millions of measurements from the human body. With current state of the art automatic analysis methods, these detailed datasets are typically heavily reduced to a few outputs of a priori specified measurements of interest (e.g. volumes, areas, diameters, average/maximum tracer concentrations etc.) [1,2,3,4,5,6]. This reduction is a major limitation in the analysis, especially of systemic and potentially systemic diseases where the whole body is affected, such as cancer and diabetes. Furthermore, statistical interaction with non-imaging data (e.g. hypothesis testing) can also only be assessed on these a priori specified measurements.

Large scale whole-body MR data is being collected together with non-imaging data in Uppsala by us, e.g., POEM (www.medsci.uu.se/poem, PI: L. Lind), EpiHealth (www.epihealth.se, PI: L. Lind) and UCAN (www.u-can.uu.se, co-PI: H. Ahlström), and elsewhere, e.g., UK Biobank (www.ukbiobank.ac.uk, up to 100 000 individuals will be scanned) and German Cohort Biobank (www.nationale-kohorte.de, up to 30 000 volunteers will be scanned). However, tools for holistic analysis of this data have, to our knowledge, not been published.

Here, we present an image analysis concept, including image registration, which allows statistical and holistic analysis of whole-body image data [7], with example analyses on whole-body image data from the POEM cohort, which aims to investigate a population-based sample of 50-year old inhabitants of the city of Uppsala invited in a random manner to study pathophysiological links between obesity and vascular dysfunction and future cardiovascular disorders, and from a weight loss and gastric bypass study.

The focus of this work is whole body image registration with spatially varying tissue constraints. By utilizing tissue-specific elasticity constraints in the image registration under the assumption that bone is relatively rigid and has low inter-subject variation, lean tissue is less rigid and has higher inter-individual variation, and adipose tissue is highly elastic and has high inter-subject variation, all whole-body images are deformed by image registration to a common coordinate system. The so-obtained point-to-point correspondences are utilized in the statistical analysis.

Medical image registration is often considered a challenging task due to, e.g., imperfections in the image data and varying morphology. Therefore, a multitude of methods designed for different body regions based on different theoretical foundations, including parametric, non-parametric, landmark-based image registration, etc. with different transformation models, matching criteria and optimization methods have been developed [8,9,10].

Whole-body water-fat MRI image registration methods related to the one presented here includes the method based on so-called morphons presented by Karlsson et al. [2]. They use the registration method together with a multi-atlas approach to achieve a stable fat and muscle segmentation and quantification. Methods for spatially varying elasticity constraints in image registration are often based on putting constraints on the parametric deformation field [11,12,13] and, for CT image data, on the assumption that tissue with low Hounsfield units are more elastic than tissue with high Hounsfield units. The latter idea has been used in, e.g., deformable registration of thorax CT volumes [11,12] and registration of whole body mice μCT volumes [14,15,16], where the high contrast between bone and other tissue is utilized by registering segmented bone as an initial step.

Work related to the statistical analysis presented here includes VBM (voxel based morphometry, available in e.g. the software SPM), which is a concept for processing of neuroimaging MRI data [17]. By image registration to a common standardized volume and segmentation of different tissues (grey matter, white matter, etc.), group comparisons and correlation analysis are performed by statistics on morphological or functional data. SPM is a concept developed for brain imaging studies. A potential alternative to the statistical analysis presented in this paper is deep-learning, which has been used for, e.g., predicting missing data in multi-modal analysis [18] and in structural and functional brain imaging data [19].

Related holistic analysis methods includes Radiomics, which is an initiative to use radiology medical imaging to monitor the development and progression of cancer or its response to therapy providing a comprehensive quantification of the tumor phenotype [1]. Other holistic, non-imaging based, methods which have successfully been used in medicine for analysis of large amounts of collected data are proteomics [20], genomics [21] and metabolomics [22].

The presented concept differs from the ones found in literature in the robust whole-body image registration approach and in that it is holistic in the following three aspects:

- The whole human body is analyzed, as opposed to ROI-based methods where information extracted from pre-defined, pre-segmented regions is used in the analysis.
- All collected image data is used in the analysis, as opposed to ROI-based methods and most deep-learning methods, in which the amount of image data is typically reduced by downsampling.
- Relationships to all other collected non-imaging patient information can be used in the analysis.

**Fig 1:**
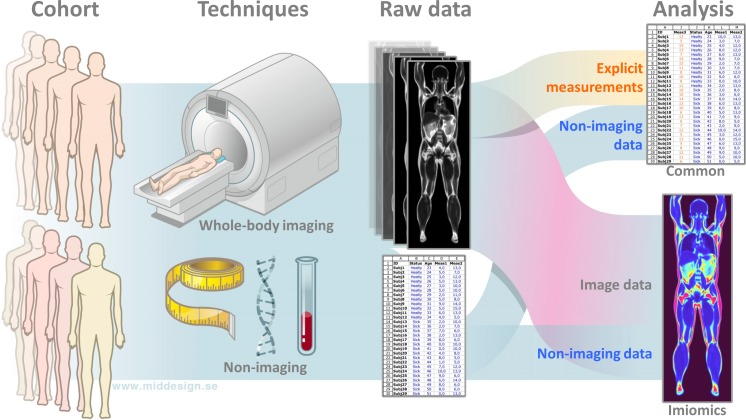
Illustration of Imiomics compared to a standard analysis approach. The standard approach analyses only a small amount of the collected imaging information using explicit measurements. Imiomics uses all collected imaging information and allows analyses of relationships to non-imaging data. In addition to more efficient data usage this allows both untargeted and targeted statistical analysis in the whole-body region, i.e. completely new types of imaging studies.

We have termed the concept *Imiomics*. An illustration of the concept, and how it differs from standard analysis methods, is shown in Fig 1.

## Methods

**Fig 2:**
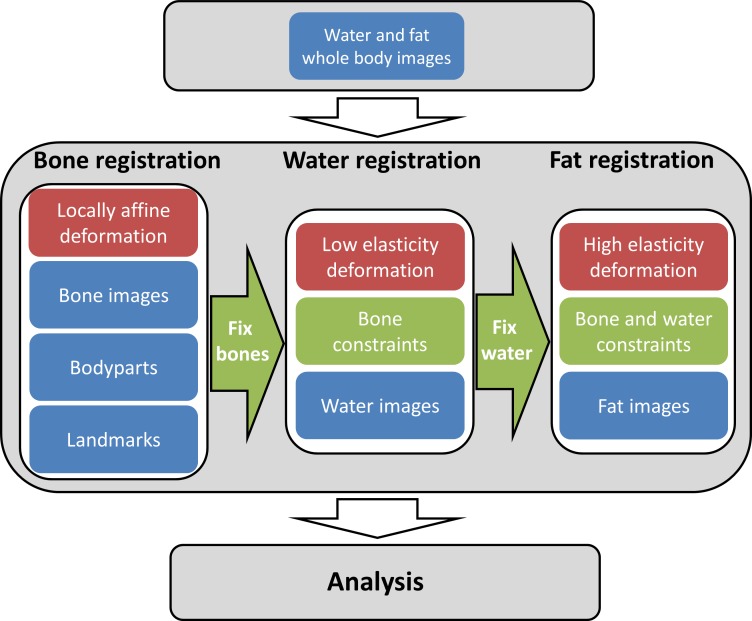
Flowchart of key steps in the registration process. Blue: Image data used, Green: constraints used in the registration.

In image registration, a source (moving) image is deformed to match a reference (fixed) image by a deformation field. Our proposed method utilizes a tissue-specific handling of bone, lean tissue, and adipose tissue. The degree of elasticity of the deformation required to align two images tends to differ between these different tissue types. This prior knowledge is utilized by performing the image registration of the different tissues sequentially, using appropriate registration parameters for each tissue. This process, described in detail below, has been implemented and evaluated on MRI fat/water separated image data. Fig 2 shows a flowchart of the image registration process.

### Input data and preprocessing

The evaluation and example Imiomics analyses were performed on a subset of whole-body MR images from the POEM cohort (n = 128) and a subject from a weight loss and gastric bypass study. Ethical approval for the study was obtained from the Regional Ethical Review Board in Uppsala, Sweden (Approval numbers: Uppsala Dnr 2009/057 and Dnr 2012/143), and written consent was obtained from all subjects. All subjects were imaged on a 1.5T clinical MR system (Philips Achieva, Philips Healthcare, Best, Netherlands) in supine position using the body coil and a whole body water-fat imaging protocol that used a spoiled 3D multi gradient echo sequence. Scan parameters were: TR/TE1/ΔTE = 5.9/1.36/1.87 ms, 3 unipolar echoes, flip angle 3 degrees. Imaged field of view (FOV) 530×377×2000 mm^3^, reconstructed voxel size 2.07×2.07×8.0 mm^3^ in left-right×anterior-posterior×foot-head directions. The imaging protocol and the water-fat image reconstruction method ASR, which is robust to B_0_ field inhomogeneities, have previously been described [5,23].

**Fig 3:**
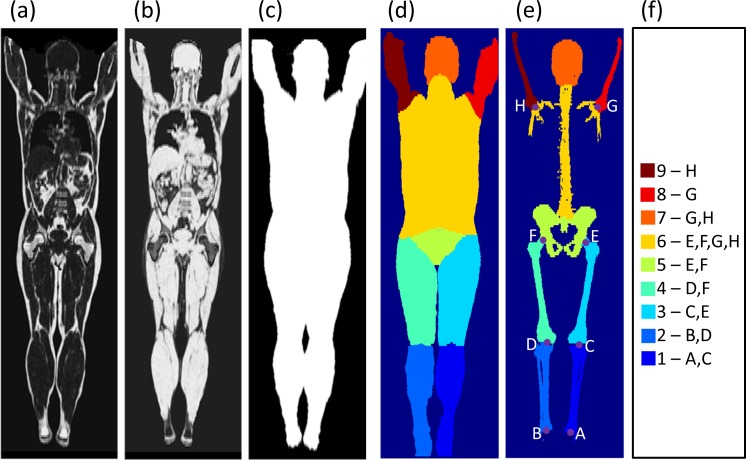
Input image examples. (a): Absolute fat content, *I*_*FAT*_. (b): Absolute water content *I*_*WATER*_. (c): Body mask *I*_*BODY*_. (d): Bodypart segmentation. (e): Bone segmentation *I*_*BONE*_, bone segments and anatomical landmarks. (f): Body part and bone segment color coding and landmarks used for each bodypart/bone section in the registration procedure.

MRI images with absolute fat and water content, denoted *I*_*FAT*_ and *I*_*WATER*_, and body masks, denoted *I*_*BODY*_, were used as input images. The images were corrected for intensity inhomogeneities by slice-wise normalization of intensity values in the foot-head direction (to avoid discontinuities between adjacent axial slices) and simultaneous correction (SIM) [24]. Example images are shown in Fig 3. The body mask was extracted from the image data by intensity thresholding of the sum of the fat and water images followed by morphological operations and region growing.

In addition to the input images *I*_*FAT*_, *I*_*WATER*_, and *I*_*BODY*_, a bodypart image, a bone image and eight anatomical landmarks, see Fig 3, were utilized in the image registration method. These can be obtained by, e.g., semi-automatic methods, morphological filtering or multi-atlas methods. In the present implementation, the bodypart segmentation and landmark extraction were obtained by a multi-atlas approach: Six whole-body volume images (from three male and three female subjects) were selected based on BMI, by including subjects with low (male: 20.3 kg/m^2^, female: 19.0 kg/m^2^), medium (male: 25.9 kg/m^2^, female: 26.2 kg/m^2^) and high (male: 36.2 kg/m^2^, female: 39.9 kg/m^2^) BMI. The semi-automatic segmentation of *I*_*BODY*_ into nine bodyparts (lower legs, upper legs, pelvis, torso, head, upper arms) using SmartPaint [25] and manual extraction of eight landmarks (ankle joint, knee joint, femoral head, humeral head) were carried out on these six whole-body volume images. These segmentations and landmarks were deformed to all input images by a rough image registration (Elastix with an affine pre-registration, B-Spline grid, normalized correlation and final grid spacing 32 mm) using *I*_*FAT*_ and *I*_*BODY*_ by a standard multi-atlas technique (majority voting and mean landmark position, respectively) [26]. Male and female subjects were treated separately in the multi-atlas process.

Bone images were obtained by extracting regions with low signal in water-fat separated MRI (air and bone give no signal in water-fat separated MRI). From this, a segmented skeleton was obtained by removing segmented lung and abdomen and by utilizing filtering techniques [27]. The bone segmentation result was further refined by a majority-voting multi-atlas technique as described above. The bone image is 1 for bone voxels and 0 otherwise.

### Image registration

An image is a mapping *I*: Ω → ℝ, here Ω ⊂ ℤ^3^. In image registration, a transformation *T*(*x*) = *x* + *u*(*x*) that makes the deformed moving (source) image *I*_*M*_(*T*(*x*)) spatially aligned to the fixed (reference) image *I*_*F*_(*x*) is sought. The transformation is typically computed by minimizing a cost function. The cost function can for example be sum of squared intensity differences, mutual information or correlation.

The resulting optimization problems are commonly solved using local search techniques. To avoid poor local minima, “coarse-to-fine” hierarchical strategies are typically used [28], where the calculation of a fine scale deformation is preceded by a matching on a more global scale. The registration is thus formulated as a composition of multiple transformations, i.e., *T* = *T*^*n*^ ° *T*^*n*−1^ ° … ° *T*^1^, of increasing fidelity.

A parametric image registration approach, realized by using the Elastix software [29], was used. In the implementation, the deformation field, parameterized by a B-spline representation, was achieved by energy minimization of a metric, i.e., weighted terms of energy functions and regularization terms. Essential steps in a parametric approach to image registration are choice of cost-functions, parameterization of the deformation field, optimization method to find the optimal parameters and interpolation methods.

The cost functions used here were

- Sum of squared differences: $S_{SSD}(T;I_{F},I_{M})=\frac{1}{\left|\Omega_{F}\right|}\sum_{x_{i}\in \Omega_{F}}{\left(I_{F}(x_{i})-I_{M}(T(x_{i}))\right)}^{2}$, where Ω_*F*_ is the fixed image domain.
- Landmark regularization: Anatomical landmarks can be used to guide the registration process. Here, the following cost function was used: $S_{LM}(T,{\left(x_{Fi},x_{Mi}\right)}_{i=1}^{P})=\frac{1}{P}\sum_{x_{Fi}}\left\|x_{Mi}-T(x_{Fi})\right\|$, where *P* is the number of landmarks, *x*_*Mi*_ is landmark *i* in the moving image and *x*_*Fi*_ is landmarks *i* in the fixed image.
- Multi-band cost function: To allow multiple image input, a cost function that can handle multiple images is needed. Here, this was accomplished by using the following form of the cost function: $C(T,I_{F},I_{M})=\frac{1}{\sum_{i=1}^{N}\omega_{i}}\sum_{i=1}^{N}\omega_{i}S_{i}(T,I_{F}^{i},I_{M}^{i})$, where *N* is the number of images and *ω* are the relative weights of the individual cost functions.

The B-spline parameters are denoted *μ* and the optimization problem to solve can be written ${\hat{T}}_{\mu }={\mathrm{argmin}}_{T_{\mu }}C(T,I_{F},I_{M})$. A stochastic gradient descent optimization method is used due to its computational efficiency [30]. To get a stable optimization process, a multi-resolution approach based on spatial down-sampling and Gaussian scale-space is also used [29].

### Detailed image registration process

The inter-subject morphological variation in bone, lean tissue (water signal), and adipose tissue (fat signal) is assumed to be relatively low, medium and high, respectively. The image registration process is divided into three steps, where different tissues are registered based on their morphological variation, starting with the tissue with lowest shape variation, bone.

The cost functions used in the individual steps of the registration procedure are weighted sums of cost functions. Input to the cost functions are pairs of moving image (*I*_*M*_) and fixed image (*I*_*F*_) and/or sets of landmarks. Cost functions used in the different steps are described below.

#### Step 1, articulated, piece-wise affine, registration of bone sections

The input data to the bone registration is eight landmarks (ankle joints, knees, hip joints and shoulder joints), segmented bone and nine bodyparts (lower legs, upper legs, pelvis, torso, head and upper arms). To obtain a reliable bone registration, an initial deformation is obtained by a similarity transform, i.e., *T*_*μ*_(*x*) = *sR*(*x* − *c*) + *t* + *c*, where *R* is a rotation matrix, *c* is the center of rotation, *s* is a scale factor and *t* is a translation vector. The initial transform is obtained by Procrustes analysis of the eight pairs of landmarks, i.e. a minimization of the mean squared distance of point pairs under rotation and scaling. The initial transform is followed by a piecewise affine transform for each of the segmented and labeled bone sections together with the one, two or four closest landmarks (see Fig 3). For the spine, the four closest landmarks are used and a slightly elastic deformation is allowed to compensate for different articulations of the spine.

In more detail, a separate cost function is optimized for each bone section:

$$C(T_{\mu },I_{F},I_{M})=\omega_{bone,1}S_{SSD}\;(T_{\mu };DT(I_{BONE,F}),DT(I_{BONE,M}))+\omega_{bone,2}S_{LM}(T_{\mu },{LM}_{k}),$$ (1)

where the distance transform *DT*(*I*(*x*)) = min_*y*:*I*(*y*) = 1_ |*x* − *y*| at each background voxel equals the distance to the closest object voxels and the pairs of landmarks ${LM}_{k}={\left(x_{Fi},x_{Mi}\right)}_{i=1}^{P}$ correspond to the landmarks given in Fig 3, in the fixed and moving images, is optimized. To optimize for only one bone section, the image domain Ω_*F*_ in the optimization used here is given by the corresponding bodypart in *I*_*BODY*_.

In Eq (1), *T*_*μ*_ is an affine transform for all objects except the spine (if *k* ≠ 5), for which a deformable transform (B-spline grid, spacing 64 mm, Gaussian downsampling with factor 4,2,1 in each dimension) is used.

A set of corresponding point pairs in the fixed and moving images, *LM*_*BONE*_, is computed for each bone section. This is done by randomly placing 1000 uniformly distributed points in each bone segment fixed image and map the points to the moving image by the obtained deformation. These point pairs will be used to regularize the deformation in the preceding steps.

#### Step 2, low elasticity registration of water images with constraints on bone

The fixed and moving water images are registered by optimizing the following cost function:

$$C(T_{\mu },I_{F},I_{M})=\omega_{wat,1}S_{SSD}(T_{\mu };\;I_{WATER,F},\;I_{WATER,M})+\omega_{wat,2}S_{LM}(T_{\mu },{LM}_{BONE}),$$

where *T*_*μ*_ is a deformable transform (B-spline grid, spacing 64mm, Gaussian downsampling with factor 4,2,1 in each dimension). This gives the approximate correct position of lean tissue, muscles etc.

In total 4000 pairs of matching points from the so-obtained water registration, *LM*_*WATER*_ are computed in voxels with high (> 50%) water signal.

#### Step 3, high elasticity registration of fat images with constraints on bone and water

The fixed and moving fat images are registered by optimizing the following cost function:

$$C(T_{\mu },I_{F},I_{M})=\omega_{fat,1}S_{SSD}(T_{\mu };\;I_{FAT,F},\;I_{FAT,M})+\omega_{fat,2}S_{SSD}(T_{\mu };\;I_{BODY,F},\;I_{BODY,M})+\omega_{fat,3}S_{LM}(T_{\mu },{LM}_{BONE})+\omega_{fat,4}S_{LM}(T_{\mu },{LM}_{WATER}),$$

where *T*_*μ*_ is a deformable transform (B-spline grid, spacing 32mm, Gaussian downsampling with factor 4,2,1 in each dimension)

### Evaluation of the registration method

Assume that a moving/source volume *A* is deformed to a fixed/reference volume *B* by a transformation *T*_*A*→*B*_(*x*) = *x* + *u*_1_(*x*) and that volume B is deformed to A by the transformation *T*_*B*→*A*_(*x*) = *x* + *u*_2_(*x*). Then, ideally, the composition of the two transformations, *T*_*B*→*A*_°*T*_*A*→*B*_(*x*) = *T*_*B*→*A*_(*T*_*A*→*B*_(*x*)), is the identity transform. In a real-world registration problem, this ideal situation is very unlikely to happen, due to interpolation effects, intensity variations, artefacts, etc. However, the closer the composition of the two transforms is to the identity transform, the better the registration result is. The evaluation method is denoted inverse consistency [31] and is computed as

- Vector magnitude error (VME): $\frac{1}{\left|\Omega_{B}\right|}\sum_{x\in I_{B}}\left|x-T_{B\to A}{}^{\circ}T_{A\to B}(x)\right|$ and
- Intensity magnitude error (IME): $\frac{1}{\left|\Omega_{B}\right|}\sum_{x\in I_{B}}\left|I_{B}(x)-I_{B}(T_{B\to A}{}^{\circ}T_{A\to B}(x))\right|$.

The optimal value for both VME and IME is 0. In addition to VME and IME, the Dice coefficients of fat and water content images thresholded at 50% before and after these composed deformations are used for evaluating the image registration method.

For the evaluation and initial Imiomics example cross-sectional analyses, one female (BMI 25.5 kg/m^2^) and one male (BMI 25.9 kg/m^2^) ‘mean’ person, which define the common coordinate system, were selected from MRI scans of 68 female and 60 male from the POEM cohort. The selection of the mean subjects was based on having a BMI (and other non-imaging parameters) close to the average.

### Imiomics example analyses

A number of example analyses of the proposed Imiomics methodology were also performed by the point-to-point correspondences obtained by the image registration. The analysis uses point-wise fat content values obtained by the water-fat separated MRI images and point-wise local expansion/compression computed from the deformation field obtained by the image registration. Point-wise local expansion is used for local tissue volume analysis.

No corrections for multiple tests were used in the Imiomics example analyses.

#### (i) Whole body imaging atlas

A whole body imaging atlas is obtained by deforming all available images to the common coordinate system. The statistical atlas is an image where each voxel holds a so-obtained distribution of intensities in each voxel.

#### (ii) Anomaly detection

By deforming a new image to the common coordinate system, point-wise statistical comparison of image intensity values can be compared to the statistical atlas by statistical tests. In this way, regions with non-normal intensity values can be detected by deviation from the values in the statistical atlas, which holds information about the expected, normal values. This procedure results in an image with P-values, a P-map.

#### (iii) Group comparison

In this analysis, the point-wise statistical analysis is carried out on different groups of subjects. With all images in the common coordinate system, the different groups have an intensity distribution in each voxel. Group comparisons are performed by statistical tests on significant differences between these distributions. The output is again a P-map.

#### (iv) Correlation analysis

In the proposed correlation analysis, the correlation between the intensity value that each subject has in each voxel and a non-imaging parameter is computed. This results in an image with correlation values in each voxel, so-called r-maps.

#### (v) Longitudinal analysis

The longitudinal analysis presented here is obtained by point-wise differences in intensity values from different subjects, all deformed to the common coordinate system,

## Results

In this section, we present image registration evaluation results and Imiomics example analyses obtained by using the image registration method.

### Evaluation of image registration

**Fig 4:**
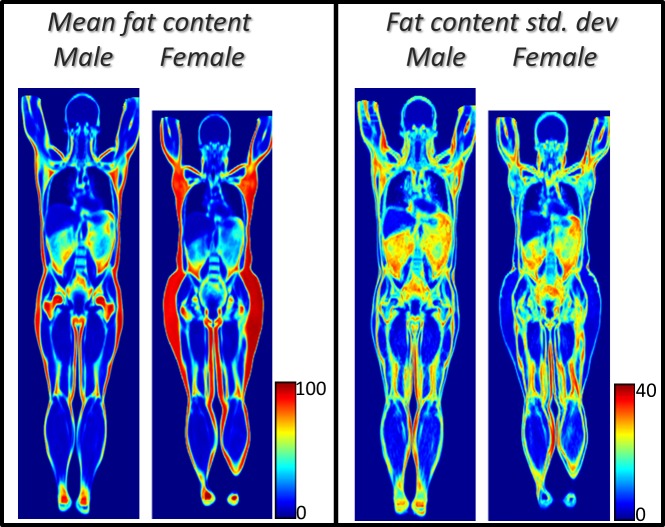
Preliminary whole body imaging atlas. Coronal slices of preliminary whole body imaging atlas—the mean and standard deviation of absolute fat content in male subjects (n = 60) and in female subjects (n = 68), from the POEM study.

Mean and standard deviation of fat content intensities obtained by deforming all volume images to a common coordinate system by the image registration method are illustrated in Fig 4.

In the inverse consistency evaluation, all female (male) subjects *A*_*i*_ were registered to a female (male) mean volume *B* and the inverse consistency was computed on the transformations given by $T_{A_{i}\to B}{}^{\circ}T_{B\to A_{i}}$ and $T_{B\to A_{i}}{}^{\circ}T_{A_{i}\to B}$ by using the above described image registration method. The mean and standard deviation of IME and VME are listed in Table 1. The Dice coefficient values obtained by comparing segmented fat and water images deformed by such compositions of deformations are listed in Table 2.

**Table 1:** Inverse consistency. Inverse consistency values for MRI scans from the POEM cohort (n = 68 for female and n = 60 for male) for the bodyparts shown in Fig 3. IME is computed on fat content (%) and VME is vector magnitude (mm). The mean and standard deviation IC values of transformations given by $T_{A_{i}\to B}{}^{\circ}T_{B\to A_{i}}$ and $T_{B\to A_{i}}{}^{\circ}T_{A_{i}\to B}$ (see text) are shown.

Body part	1	2	3	4	5	6	7	8	9
Female IME	6.6 ±1.5	6.1 ±1.5	7.2 ±1.4	7.0 ±1.3	7.7 ±1.4	8.1 ±1.2	4.0 ±1.1	12.1 ±1.8	9.7 ±1.8
Female VME	2.3 ±0.8	2.1 ±0.7	4.1 ±1.0	4.2 ±1.1	4.0 ±1.1	4.6 ±1.1	1.9 ±0.5	6.3 ±1.8	4.0 ±1.7
Male IME	7.6 ±1.8	7.6 ±1.7	6.9 ±1.4	6.4 ±1.4	8.3 ±1.8	9.1 ±1.3	5.2 ±1.5	11.4 ±2.3	11.2 ±2.7
Male VME	3.0 ±1.5	3.4 ±1.7	3.2 ±0.8	3.2 ±0.8	3.6 ±1.0	4.9 ±0.9	2.7 ±1.2	6.0 ±3.0	5.9 ±4.6

**Table 2:** Dice values. Dice values obtained by composition of deformation fields for MRI scans from the POEM cohort (n = 68 for female and n = 60 for male) for the bodyparts shown in Fig 3. The fat and water (wat) content images are thresholded at 50% before and after the composed deformation. The Dice coefficient values are computed on these thresholded binary images. The table holds the mean and standard deviation Dice values.

Body part	1	2	3	4	5	6	7	8	9
Female Dice fat	0.85±0.05	0.86 ±0.05	0.93 ±0.02	0.94 ±0.01	0.91 ±0.02	0.88 ±0.03	0.79 ±0.07	0.82 ±0.06	0.85 ±0.08
Female Dice wat	0.94 ±0.02	0.94 ±0.02	0.94 ±0.01	0.94 ±0.01	0.92 ±0.02	0.91 ±0.02	0.96 ±0.02	0.88 ±0.04	0.91 ±0.05
Male Dice fat	0.87 ±0.04	0.85 ±0.04	0.92 ±0.02	0.93 ±0.02	0.90 ±0.02	0.90 ±0.02	0.78 ±0.06	0.87 ±0.04	0.87 ±0.03
Male Dice wat	0.95 ±0.02	0.93 ±0.02	0.96 ±0.01	0.96 ±0.01	0.91 ±0.02	0.90 ±0.02	0.96 ±0.02	0.94 ±0.02	0.95 ±0.02

### Imiomics example analyses

#### (i) Whole body imaging atlas

Statistical image data representations showing pointwise mean, and standard deviation, of fat content of male and female subjects is shown in Fig 4. Note the sharp borders in the mean images and the relatively low standard deviations.

#### (ii) Anomaly detection

**Fig 5:**
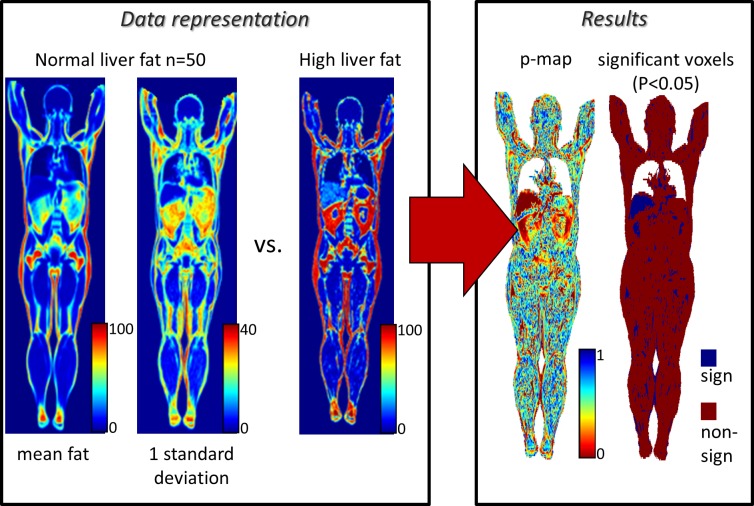
Anomaly detection. The anomaly detection was performed by comparing intensities of one male subject with high liver fat content to a preliminary whole body imaging atlas, of subjects with normal liver fat, that holds pointwise distributions of fat content of 50 male subjects. The whole-body imaging atlas is visualized by the mean value and standard deviation.

Example of high liver fat anomaly detected by deviation of a subject from a group with normal liver fat content (<10%) is shown in Fig 5. This example anomaly detection indicates low P-values where expected, i.e. in the abdominal adipose tissue and in the liver, respectively.

#### (iii) Group comparison

**Fig 6:**
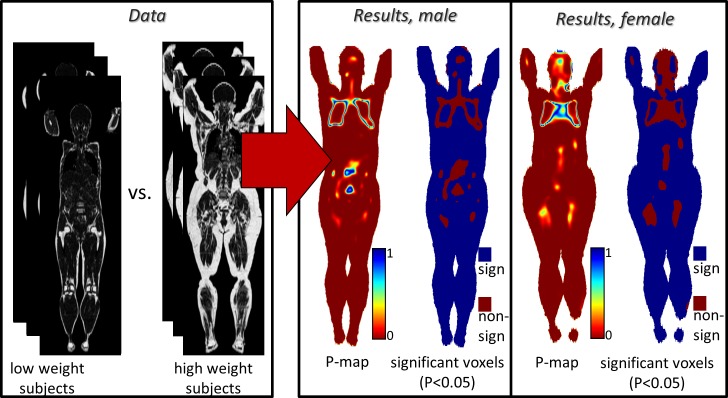
Group comparisons. The point-by-point P-values (P-maps) of local tissue volume were obtained by two-tailed t-tests between low weight subjects and high weight subjects (20 men and 23 women).

Fig 6 shows pointwise comparisons of local tissue volume between a group of high weight subjects and a group of low weight subjects. Significant differences of local tissue volume (size) were observed between the two groups in almost the whole body.

#### (iv) Correlation analysis

**Fig 7:**
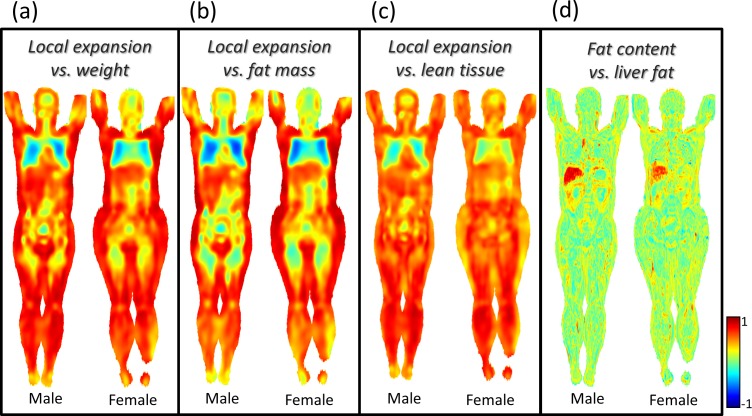
Correlation analysis. Coronal slices of maps with point-wise correlation (r-value) between (a) local tissue volume and weight, (b) local tissue volume and total body fat mass measured by bioimpedance analysis (BIA), (c) local tissue volume and lean tissue (weight minus total body fat mass) and (d) fat content and mean liver fat content. These example correlation analyses are based on 60 male and 68 female subjects.

Fig 7 shows correlation analyses between image data and the continuous variables (biomarkers) weight, total fat mass (using a bioimpedance scale, Tanita, Japan), lean tissue (difference between weight and total fat mass) and average liver fat content (explicitly measured from a separate liver scan).

Fig 7(A) shows strong positive correlations between local expansion and weight in adipose tissue while correlations in other tissues are weaker.

Fig 7(B) shows strong positive correlations between local expansion and total fat mass in adipose tissue while, again, correlations in other tissues are weaker. The expected finding in Fig 7(B) that fat volume correlates with total body fat mass was confirmed by explicitly measured volumes from MRI (adipose tissue: r = 0.95, P<0.001, lean tissue: r = -0.11, P = 0.158, leg muscle: r = -0.03, P = 0.668). The correlation map in Fig 7(B) also shows a finding that we did not foresee in the negative correlation between lung volume and body fat mass (also confirmed by explicit measurements, r = -0.38, P<0.001).

Fig 7(C) shows strong positive correlation between local expansion and total lean tissue mass in lean tissue.

Fig 7(D) shows strong positive correlation between absolute fat content and explicitly measured liver fat content in the liver.

#### (v) Longitudinal analysis

**Fig 8:**
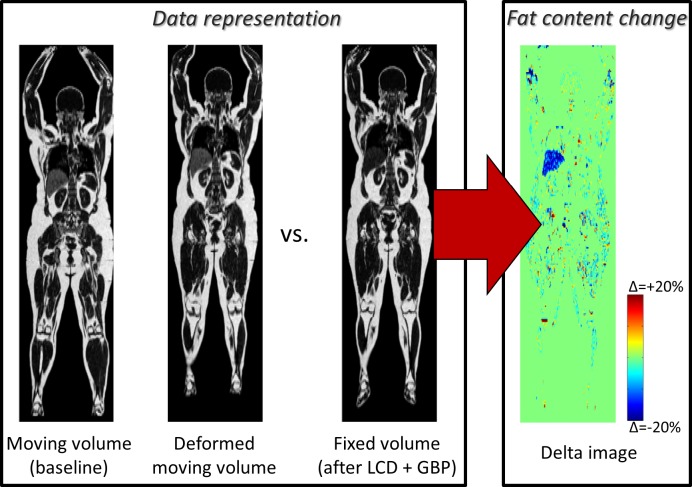
Longitudinal analysis. One coronal slice of the moving, deformed moving and fixed images of absolute fat content is shown together with a difference image between the fixed and deformed moving images. The subject underwent low calorie diet (LCD) and gastric bypass (GBP) between the moving and fixed MRI-scans. The total weight loss was 14 kg.

In Fig 8, difference in fat content before and after gastric bypass surgery for morbid obesity is shown. Note the visualization of the reduction in liver fat, already at one month after surgery.

## Discussion and conclusions

In this paper, we have introduced an image analysis concept and described and evaluated a stable image registration method, suited for the analysis. We have, by example analyses, illustrated how the concept can be used for statistical and holistic analysis of whole-body image data and for integrating non-imaging patient information.

The mean and standard deviation images in Fig 4 show sharp borders between different tissues. Relatively high standard deviation is observed in the abdomen and in the subcutaneous fat between the legs (since the legs were not always separated due to non-standardized positioning). The inverse consistency values in Table 1 and the Dice coefficient values in Table 2 show high agreement between the reference and deformed images. The IME, VME and Dice coefficient values are based on the composition of two transforms; still the VME values are in the order of the voxel size (2.07×2.07×8.0 mm^3^). The lower arms are omitted in the analyses presented here since the hand positioning in the used images was not standardized. Image artefacts introduced some errors in the analysis; artefacts due to large FOV imaging in upper arms and female thigh degraded the registration result in these regions. The higher average whole body IME and VME errors (Table 1) in males might be due to the selection of the male ‘mean’ subject, which had a less fat mass compared to the female subject (14.2kg vs 22.4kg as measured by bioimpedance analysis). High IME error was observed along tissue borders. This is caused by small tissue border mismatches and partial volume effects that result in large differences in fat content.

The requirements on the image registration method in Imiomics analyses are higher than for, for example, in multi-atlas segmentation, where labels are propagated from a number of pre-segmented images and some registration errors caused by, for example, too high or too low degree of elasticity are acceptable and handled by the multi-atlas step. Multi-atlas segmentation is used as a pre-processing step in this paper. The applications mentioned in this paper require reliable point-to-point correspondences and in our future work, we plan to further evaluate the registration method by using also other measures of registration accuracy and a comparison with publically available registration methods. Future work also includes analysis on test-retest reliability and the effect of the choice of mean person as well as correction for multiple tests taking both inter-voxel intensity redundancy and deformation field smoothness into account.

Large-scale analyses of volume images require large amounts of computer resources, both for storing the input and output data and for the computations. With the current setup, each whole body registration requires approximately one hour on a standard PC (Windows 7, Intel Core i7-3770, 3.4 GHz, 16 GB RAM). We are currently working on optimization strategies for reducing the computation time.

We have, by example applications, shown that Imiomics analyses based on the proposed image registration method give expected results for:

1. Creation of a whole body imaging atlas of normality, which can be used as a representation of normality, to which new images can be compared, for example, point-wise, tissue-wise, or organ-wise to detect anomalies.
2. Automated anomaly detection by using the whole body imaging atlas. Imiomics successfully detected the high liver fat anomaly in the example in Fig 5. This can be used to detect both morphological and image intensity anomalies. Tumors might for example be detected based on both morphology and intensity patterns (in for example PET tracer uptake images).
3. Group comparisons. The group comparison presented in Fig 6 showed, as expected, differences in local tissue volume (size) between these a group of high weight subjects and a group of low weight subjects. The significant lung volume differences is in agreement with the correlation analysis below. Group comparison analysis allows comparison of groups obtained by, for example, thresholding ordinal or continuous variables/biomarkers or by categorical variables/biomarkers
4. Correlation analysis, which can be used to detect both morphological and image intensity associations. Expected positive correlations between subcutaneous fat and both weight and total fat mass were observed in Fig 7A and 7B. The correlations in lean tissue were weaker. The negative correlations between lung volume and body fat mass in Fig 7(B) exemplifies the possibility to use Imiomics for untargeted analysis. Expected correlations in absolute liver fat content were observed in the example in Fig 7(D).
5. Longitudinal change detection and analysis. Fig 8 shows a very high reduction in liver fat between baseline and follow-up. Other examples of longitudinal analyses include disease progression, drug efficacy and e.g. weight loss where this technology allows “imaging” of changes.

Based on the inverse consistency evaluation and by the successful computation of Imiomics analyses, we conclude that the image registration method is well-suited for use in Imiomics-analyses.

Further possibilities of Imiomics include prediction analysis based on imaging data, for example future risk of type-2 diabetes, myocardial infarction, stroke or dementia predicted from image data. Also, by deforming an anatomical whole-body atlas using the methods presented here, both tissue and organ quantification or characterization can be achieved. This might for example be used to improve MR-based attenuation correction for PET-MR via separation of bone-air [32].

We conclude that Imiomics enables new types of holistic untargeted analysis, targeted analysis and analyses of relationships to non-imaging data and allows new types of research studies applied in studies of systemic and potentially systemic diseases like cancer and diabetes.

The interest in advanced image analysis methods like Imiomics is increasing with the ever-increasing amount of collected image data. Very large scale whole-body MR image data is already today being collected in several projects, as described in the introduction, opening an avenue for Imiomics analyses and its potential to improve our understanding of diseases that traditional analysis techniques cannot achieve.

## Supporting information

S1 File Data for Table 1 and Table 2. (ZIP)
